# Efeitos do Exercício Aeróbico Tardio na Remodelação Cardíaca de Ratos com Infarto do Miocárdio Pequeno

**DOI:** 10.36660/abc.20190813

**Published:** 2021-04-08

**Authors:** Lidiane M. Souza, Marina P. Okoshi, Mariana J. Gomes, Mariana Gatto, Eder A. Rodrigues, Thierres H. D. Pontes, Felipe C. Damatto, Leiliane R. S. Oliveira, Patrícia Aparecida Borim, Aline R. R. Lima, Leonardo A. M. Zornoff, Katashi Okoshi, Luana U. Pagan

**Affiliations:** 1 Universidade Estadual Paulista “Júlio de Mesquita Filho” Faculdade de Medicina de Botucatu BotucatuSP Brasil Faculdade de Medicina de Botucatu, Universidade Estadual Paulista “Júlio de Mesquita Filho” (UNESP), Botucatu, SP – Brasil.

**Keywords:** Exercício, Atividade Física, Disfunção Ventricular, Infarto do Miocárdio, Ratos, Remodelação Ventricular, Ecocardiografia/métodos, NADPH Oxidase

## Abstract

**Fundamento:**

O exercício físico tem sido considerado uma importante terapia não farmacológica para a prevenção e tratamento das doenças cardiovasculares. No entanto, seus efeitos na remodelação cardíaca leve não são claros.

**Objetivo:**

Avaliar a influência do exercício aeróbico sobre a capacidade funcional, estrutura cardíaca, função ventricular esquerda (VE) e expressão gênica das subunidades da NADPH oxidase em ratos com infarto do miocárdio pequeno (IM).

**Métodos:**

Três meses após a indução do IM, ratos Wistar foram divididos em três grupos: Sham; IM sedentário (IM-SED); e IM exercício aeróbico (IM-EA). Os ratos se exercitaram em uma esteira três vezes por semana durante 12 semanas. Um ecocardiograma foi realizado antes e após o treinamento. O tamanho do infarto foi avaliado por histologia e a expressão gênica por RT-PCR. O nível de significância para análise estatística foi estabelecido em 5%.

**Resultados:**

Ratos com IM menor que 30% da área total do VE foram incluídos no estudo. A capacidade funcional foi maior no IM-EA do que nos ratos Sham e IM-SED. O tamanho do infarto não diferiu entre os grupos. Ratos infartados apresentaram aumento do diâmetro diastólico e sistólico do VE, diâmetro do átrio esquerdo e massa do VE, com disfunção sistólica. A espessura relativa da parede foi menor no grupo IM-SED do que nos grupos IM-EA e Sham. A expressão gênica das subunidades NADPH oxidase NOX2, NOX4, p22^phox^ e p47^phox^ não diferiu entre os grupos.

**Conclusão:**

Infarto do miocárdio pequeno altera a estrutura cardíaca e a função sistólica do VE. O exercício aeróbico tardio pode melhorar a capacidade funcional e a remodelação cardíaca por meio da preservação da geometria ventricular esquerda. A expressão gênica das subunidades da NADPH oxidase não está envolvida na remodelação cardíaca, nem é modulada pelo exercício aeróbico em ratos com infarto do miocárdio pequeno.

## Introdução

Doenças cardiovasculares estão entre as principais causas de mortes no mundo, sendo o infarto do miocárdio (MI), a principal causa de morbidade e mortalidade.^1^

O IM agudo leva à remodelação cardíaca, definida como anormalidades na expressão do genoma resultando em mudanças moleculares, celulares e intersticiais que se manifestam clinicamente como alterações no tamanho, forma e função do coração.^2^ O estresse oxidativo, caracterizado por desequilíbrio entre a produção de espécies reativas de oxigênio e sistemas antioxidantes, é frequentemente observado na remodelação cardíaca.^3^ O complexo nicotinamida adenina dinucleotídeo fosfato (NADPH) oxidase, uma importante fonte de produção de espécies reativas celulares de oxigênio,^4^ costuma aumentar após o IM.^5^

Nas últimas décadas, o exercício físico emergiu como uma importante terapia não farmacológica para prevenir e tratar várias doenças cardiovasculares.^6^ O exercício aeróbico tem sido o foco de muitos estudos sobre a atenuação da remodelação cardíaca induzida por IM, melhora da capacidade funcional e qualidade de vida.^7^^–^^10^

Modelos animais de IM são amplamente usados para estudar a fisiopatologia e o tratamento da remodelação cardíaca. A maioria dos estudos que avaliam os efeitos do exercício nas alterações cardíacas pós-infarto do miocárdio utilizou roedores com grandes áreas de infarto, geralmente mais de 30% da área total do ventrículo esquerdo (VE).^8^^,^^11^^–^^14^ No entanto, ainda não está claro se o exercício aeróbico é útil para atenuar as alterações cardíacas após infarto do VE de tamanho menor. Neste estudo, objetivamos avaliar a influência do exercício físico aeróbico na capacidade funcional, estruturas cardíacas, função do VE e expressão do gene da subunidade NADPH oxidase em roedores com IM pequeno.

## Materiais e Métodos

### Animais experimentais

Ratos Wistar machos pesando 200-250 g foram adquiridos no Biotério Central da Faculdade de Medicina de Botucatu, UNESP. Todos os animais foram mantidos em sala com temperatura controlada de 24 ± 2 °C e submetidos ao ciclo claro/escuro de 12 horas em gaiolas coletivas (três por gaiola). Comida e água foram fornecidas *ad libitum*.

Todos os experimentos e procedimentos foram aprovados pelo Comitê de Ética em Experimentação Animal da Faculdade de Medicina de Botucatu, UNESP, SP, Brasil, que segue as diretrizes estabelecidas pelo Guia para o Cuidado e Uso de Animais de Laboratório publicadas pelo US National Institutes of Health e pelo Colégio Brasileiro de Experimentação Animal (protocolo número 1237/2017).

O IM foi induzido ligando-se a artéria coronária descendente anterior esquerda por um método previamente descrito.^3^^,^^14^ Resumidamente, 60 ratos foram anestesiados com cetamina (60 mg/kg) e cloridrato de xilazina (1 mg/kg) e submetidos à toracotomia lateral esquerda. Após exteriorização do coração, o átrio esquerdo (AE) foi retraído para facilitar a ligadura da artéria coronária com fio de mononáilon 5-0 entre a via de saída do pulmão e o AE. O coração foi então recolocado no tórax, os pulmões inflados com pressão positiva e a toracotomia fechada. Quinze animais com operação simulada foram usados como controles.

Três meses depois, os ratos que sobreviveram foram submetidos a um ecocardiograma transtorácico e teste ergométrico e, então, divididos em três grupos: Sham (n=15); IM sedentário (IM-SED, n=22) e IM exercício aeróbico (IM-EA, n=21) por três meses. Dezessete ratos infartados (28%) morreram durante a cirurgia ou no período pós-operatório. Os resultados iniciais do ecocardiograma foram usados para assegurar que os grupos IM sedentário e de exercício tivessem o mesmo grau de lesão cardíaca. Ao final do período experimental, os animais foram novamente submetidos a ecocardiograma e teste ergométrico, sendo eutanasiados no dia seguinte. Estudos anteriores demonstraram que a inclusão de 10 a 15 animais por grupo é suficiente para mostrar diferenças na remodelação cardíaca ao comparar ratos infartados e Sham.^3^^,^^14^

### Teste de esforço

A capacidade funcional foi avaliada antes, 45 dias após o início do exercício e ao final do experimento. Os ratos foram submetidos a 5 min/dia de adaptação ao ambiente de teste por uma semana antes da avaliação. Cada animal foi testado individualmente. O teste consistiu em um aquecimento inicial de 5 minutos a 5 m/min em uma esteira. Os ratos foram então submetidos a exercícios a 8 m/min, seguidos de incrementos de 3 m/min a cada 3 minutos, até a exaustão. A exaustão foi determinada quando o animal se recusou a correr mesmo após a estimulação elétrica ou foi incapaz de coordenar os passos.^15^^,^^16^ A velocidade máxima de corrida foi registrada e a distância total, calculada. Os resultados do teste de exercício de treinamento de 45 dias foram usados para ajustar a intensidade do exercício.

### Protocolo de treinamento de exercício

O exercício foi realizado em esteira, três dias/semana, durante três meses. Houve um período de adaptação, com aumento gradativo da velocidade e da duração do exercício. A velocidade da 1ª à 5ª semana foi de 5, 7,5, 10, 12 e 15 m/min. A duração do exercício da 1ª à 5ª semana foi de 10, 15, 25, 30 e 40 minutos. A partir da 6ª semana, cada sessão consistiu em 40 minutos de corrida a 60% da velocidade máxima alcançada no teste de exercício em esteira. O protocolo foi adaptado de Moreira et al.^17^ Após 45 dias de treinamento físico aeróbico, os animais tiveram sua performance de corrida reavaliada para ajuste da intensidade do exercício.

### Ecocardiografia

As estruturas cardíacas e a função do VE foram avaliadas por ecocardiograma transtorácico e Doppler tecidual usando um ecocardiógrafo disponível comercialmente (General Electric Medical Systems, modelo Vivid S6, Tirat Carmel, Israel) equipado com um transdutor multifrequência de 5–11,5 MHz, conforme descrito anteriormente.^18^^–^^20^ Os animais foram anestesiados com cetamina (50 mg/kg) e cloridrato de xilazina (1 mg/kg *i.p.*) e colocados em decúbito lateral esquerdo. Todas as estruturas cardíacas foram medidas manualmente pelo mesmo observador (KO). Os resultados foram a média de pelo menos cinco ciclos cardíacos nos traçados do modo-M. As seguintes variáveis estruturais foram medidas: diâmetro do AE, diâmetros diastólico e sistólico do VE (DDVE e DSVE, respectivamente), espessura diastólica da parede posterior do VE (EDPP) e diâmetro aórtico (AO). A massa do ventrículo esquerdo (MVE) foi calculada usando a fórmula [(DDVE + EDPP + ESPP)^3^− DDVE^3^] × 1,04. A espessura relativa da parede do VE (ERP) foi calculada com a fórmula 2 × EDPP/LVDD. A função sistólica foi avaliada pelos seguintes parâmetros: encurtamento fracionário endocárdico (EFE), velocidade de encurtamento da parede posterior (VEPP), mudança de área fracionada (MAF), índice de desempenho miocárdico (índice Tei) e velocidade sistólica do anel mitral (onda S’) obtida por imagem de Doppler tecidual. A função diastólica foi analisada pelas velocidades de influxo mitral diastólica precoce e tardia (ondas E e A), razão E/A, tempo de relaxamento isovolumétrico (TRIV), velocidade diastólica precoce (E’) e diastólica tardia (A’) do anel mitral (velocidades médias aritméticas de deslocamento das paredes lateral e septal) e razão E/E’.

### Coleta de tecidos para análise

Um dia após o ecocardiograma final, os animais foram pesados, anestesiados com tiopental sódico intraperitoneal (180 mg/kg) e eutanasiados. Seus corações foram removidos por toracotomia. O pulmão, átrios e ventrículos foram dissecados e pesados. Fragmentos de VE foram congelados em nitrogênio líquido e armazenados a −80 °C para análise posterior.

### Estudo morfológico

As amostras de VE foram fixadas em solução tamponada de formalina a 10% por 24 horas, em seguida lavadas em água e transferidas para solução com etanol, conforme método previamente descrito.^21^

Para calcular o tamanho do infarto, o VE foi cortado a uma distância de 5 a 6 mm do ápice.^22^ Cortes do coração foram submetidos à coloração com picrosirius red (PSR) e examinados em um microscópio composto (Leica DM LS; Nussloch, Alemanha) acoplado a um sistema de análise de imagem computadorizado (Media Cybernetics, Silver Spring, Maryland, EUA).^23^ O tamanho do infarto foi calculado dividindo-se a soma dos comprimentos ventriculares do infarto endocárdico e epicárdico pela soma das circunferências ventriculares endocárdicas e epicárdicas totais (infarto e miocárdio viável).^14^ Os valores foram expressos em porcentagem da área total do VE. Apenas ratos com pequeno IM (<30% da área total do VE) pela avaliação histológica foram incluídos no estudo.

Os diâmetros dos cardiomiócitos foram avaliados em cortes transversais do VE corados com hematoxilina-eosina. Foi, então, medido o menor diâmetro de pelo menos 50 fibras cardíacas com o núcleo claramente identificado.^24^

### Expressão gênica de subunidades NADPH oxidase

A expressão gênica das subunidades NADPH oxidase NOX2, NOX4, p22^phox^ e p47^phox^ e os genes de referência foram analisados por Reação em Cadeia da Polimerase de Transcrição Reversa Quantitativa em Tempo Real (RT-PCR), conforme descrito anteriormente.^25^ O RNA total foi extraído de amostras do VE com TRIzol Reagente (Invitrogen Life Technologies, Carlsbad, CA, EUA) e tratado com DNase I (Invitrogen Life Technologies). Um micrograma de RNA foi transcrito reversamente usando um kit de transcrição reversa de cDNA de alta capacidade, de acordo com métodos-padrão (Applied Biosystems, Foster City, CA, EUA). Alíquotas de cDNA foram então submetidas a PCR em tempo real usando um ensaio personalizado contendo primers sense e antisense (Applied Biosystems, Foster City, CA, EUA) e sondas Taqman específicas para cada gene: NOX2 (Rn00576710 m1), NOX4 (Rn00585380 m1), p22^phox^ (Rn00577357 m1) e p47^phox^ (Rn00586945 m1). A amplificação e análise foram realizadas usando o sistema Step One Plus™ Real-Time PCR (Applied Biosystems, Foster City, CA, EUA). Os dados de expressão foram normalizados para expressões gênicas de referência: ciclofilina (Rn00690933 m1) e GAPDH (Rn01775763 g1). As reações foram realizadas em triplicata e os níveis de expressão, calculados com base no método comparativo de TC (2^−ΔΔCT^).

### Análise estatística

A normalidade dos dados foi avaliada pelo teste de Shapiro-Wilk. Comparações entre os grupos foram realizadas por análise de variância (ANOVA) unilateral, seguida do teste de Bonferroni para variáveis paramétricas, que são expressas em média ± desvio-padrão. As variáveis não paramétricas foram comparadas pelo teste de Kruskal-Wallis seguido do teste de Dunn, sendo expressas em mediana e percentis. O tamanho do infarto foi comparado pelo teste t de Student não pareado. Todas as análises estatísticas foram realizadas no software SigmaStat 12.0, com nível de significância de 5%.

## Resultados

### Grupos experimentais e parâmetros anatômicos

No início do protocolo de exercício, o grupo Sham tinha 15 animais, IM-SED tinha 22 e IM-EA tinha 21. Após análise histológica, os ratos com infarto ≥ 30% da área total do VE (9 no IM-SED e 9 no grupo IM-EA) foram excluídos do estudo. Apenas um rato do IM-SED morreu durante o protocolo de exercícios. Os parâmetros anatômicos são mostrados na Tabela 1. O peso corporal final não diferiu entre os grupos. Os pesos dos átrios e do ventrículo direito (VD) foram maiores no IM-EA do que no grupo Sham. Não foram encontradas diferenças entre os grupos IM-EA e IM-SED.

**Tabela 1:** Dados anatômicos

	SHAM (n=15)	IM-SED (n=12)	IM-EA (n=12)
PC (g)	536 ± 29,7	537 ± 66,8	529 ± 44,7
VE (g)	0,90 (0,87-0,97)	0,99 (0,93-1,03)	0,99 (0,90-1,11)
VE/PC (g/kg)	1,73 ± 0,10	1,90 ± 0,19	1,88 ± 0,23
VD (g)	0,23 ± 0,03	0,26 ± 0,04	0,29 ± 0,05*
VD/PC (g/kg)	0,43 ± 0,05	0,48 ± 0,07	0,54 ± 0,08*
Peso atrial (g)	0,10 (0,08-0,11)	0,13 (0,10-0,13)	0,13 (0,11-0,14)*
Atrial/PC (g/kg)	0,19 (0,15-0,22)	0,22 (0,19-0,24)	0,27 (0,22-0,28)*
Pulmão/PC (g/kg)	3,60 (3,19-3,70)	3,43 (3,09-3,72)	3,66 (3,58-4,13)

Dados expressos em média ± desvio-padrão ou mediana e percentis. IM-SED: infarto do miocárdio sedentário; IM-EA: infarto do miocárdio e exercício aeróbico; n: número de animais; PC: peso corporal; VE: peso do ventrículo esquerdo; VD: peso do ventrículo direito. ANOVA e teste de Bonferroni ou Kruskal-Wallis e Dunn;

* p<0,05 vs. Sham.

**Figura 1:**
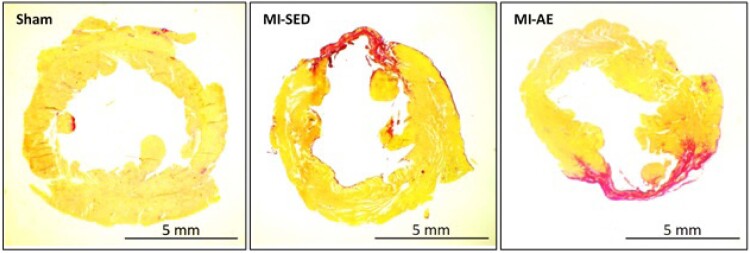
Fotos histológicas representativas de porções do ventrículo esquerdo com coloração por picrosirius red dos grupos Sham, infarto do miocárdio sedentário (IM-SED) e infarto do miocárdio e exercício aeróbico (IM-EA).

O tamanho do infarto, avaliado pela análise histológica do VE, não diferiu entre os grupos infartados (IM-SED 18,7 ± 6,41; IM-EA 23,6 ± 6,14% da área total do VE; p>0,05; Figura 1).

### Avaliação ecocardiográfica

Antes do exercício, não houve diferenças nos parâmetros ecocardiográficos entre os grupos IM-EA e IM-SED (dados não mostrados). Os dados estruturais ecocardiográficos finais estão listados na Tabela 2. Ambos os grupos infartados tinham diâmetros sistólico e diastólico, diâmetro do AE e massa do VE maiores em comparação com o grupo Sham. A espessura diastólica da parede posterior do VE foi maior no IM-EA do que no Sham, e a espessura relativa da parede foi menor no IM-SED do que nos grupos IM-EA e Sham. A função sistólica do VE é demonstrada na Tabela 3. Os grupos infartados apresentaram menor alteração da área fracionada e fração de encurtamento endocárdico, bem como maior índice Tei em relação ao Sham. A função diastólica do VE é apresentada na Tabela 4. A onda E’ (média e septal) foi menor em ambos os grupos com infarto em relação ao grupo Sham. O grupo IM-EA teve menor relação E/A em relação ao Sham. A razão E’/A’ foi menor no IM-SED do que no Sham. Não foram observadas diferenças entre os grupos de ratos infartados submetidos a exercício e sedentários.

**Tabela 2:** Dados estruturais ecocardiográficos

	SHAM (n=15)	IM-SED (n=10)	IM-EA (n=12)
FC (bpm)	267 ± 32,9	278 ± 19,7	290 ± 28,7
DDVE (mm)	8,19 ± 0,44	9,99 ± 0,81*	9,93 ± 0,98*
DSVE (mm)	4,13 (3,96-4,30)	7,16 (6,60-8,21)*	7,25 (6,73-8,16)*
EPPD (mm)	1,42 (1,40-1,45)	1,53 (1,45-1,61)	1,67 (1,58-1,85)*
AO (mm)	4,20 ± 0,15	4,12 ± 0,22	4,13 ± 0,25
AE(mm)	5,68 ± 0,42	6,71 ± 0,75*	6,97 ± 1,07*
AE/AO	1,37 (1,30-1,42)	1,64 (1,47-1,79)*	1,66 (1,47-1,82)*
DSVE/PC (mm/kg)	15,2 (14,8-16,3)	17,9 (16,9-20,3)*	18,5 (17,8-20,1)*
AE/PC (mm/kg)	10,7 ± 0,95	12,4 ± 1,42*	13,5 ± 2,46*
MVE (g)	0,84 (0,76-0,91)	1,29 (1,17-1,43)*	1,27 (1,22-1,63)*
IMVE (g/kg)	1,57 (1,46-1,70)	2,32 (2,12-2,63)*	2,44 (2,31-3,08)*
ERP	0,35 ± 0,02	0,31 ± 0,02*	0,35 ± 0,04#
% area IM	Sem infarto	26,23 ± 5,77	27,62 ± 7,67

Dados expressos em média ± desvio-padrão ou mediana e percentis. IM-SED: infarto do miocárdio sedentário; IM-EA: infarto do miocárdio e exercício aeróbico; n: número de animais; FC: frequência cardíaca; DDVE e DSVE: diâmetros diastólico e sistólico do ventrículo esquerdo, respectivamente; EPPD: espessura da parede posterior diastólica do ventrículo esquerdo; AO: diâmetro da aorta; AE: diâmetro do átrio esquerdo; PC: peso corporal; MVE: massa ventricular esquerda; IMVE: índice de massa ventricular esquerda; ERP: espessura relativa da parede. % área IM: porcentagem da área de infarto do miocárdio. ANOVA e teste de Bonferroni ou Kruskal-Wallis e Dunn;

* p<0,05 vs Sham;

# p<0,05 vs IM-SED.

**Tabela 3:** Parâmetros ecocardiográficos da função sistólica do ventrículo esquerdo

	SHAM (n=15)	IM-SED (n=10)	IM-EA (n=12)
FEE (%)	49,7 ± 3,40	27,0 ± 5,23*	26,6 ± 7,91*
VEPP (mm/s)	42,1 ± 5,66	35,9 ± 5,37	38,7 ± 9,28
FVA (%)	67,3 ± 5,07	41,1 ± 9,95*	37,6 ± 10,5*
Tei index	0,46 ± 0,06	0,58 ± 0,12*	0,58 ± 0,15*
S’ average (cm/s)	3,55 ± 0,40	3,15 ± 0,34	3,20 ± 0,47

Dados expressos como média ± desvio-padrão. IM-SED: infarto do miocárdio sedentário; IM-EA: infarto do miocárdio e exercício aeróbico; n: número de animais; FEE: fração de encurtamento do endocárdio; FVA: fração de variação da área; VEPP: velocidade de encurtamento da parede posterior; Índice Tei: índice de desempenho miocárdico; Média S’: velocidades médias máximas de deslocamento sistólico para as paredes lateral e septal do anel mitral avaliadas por Doppler tecidual. ANOVA e Bonferroni;

* p <0,05 vs Sham.

**Tabela 4:** Parâmetros ecocardiográficos da função diastólica do ventrículo esquerdo

	SHAM (n=15)	IM-SED (n=10)	IM-EA (n=12)
Mitral E (cm/s)	77,0 (71,0-85,0)	72,5 (69,3-79,5)	75,5 (72,8-78,0)
Mitral A (cm/s)	49,1 ± 12,2	54,3 ± 11,9	59,9 ± 16,8
E/A	1,71 (1,42-1,79)	1,32 (1,26-1,49)	1,23 (1,07-1,35)*
TRIV (m/s)	26,5 ± 3,42	29,7 ± 5,75	28,0 ± 3,79
E’ média (cm/s)	4,20 ± 0,63	3,52 ± 0,62*	3,58 ± 0,50*
E’ lateral (cm/s)	4,16 ± 0,73	3,20 ± 0,56*	3,24 ± 0,74*
E’ septal (cm/s)	4,24 ± 0,61	3,84 ± 0,88	3,92 ± 0,79
E/E’ média	19,1 ± 2,65	21,8 ± 3,47	21,6 ± 2,35
A’ média (cm/s)	3,05 (2,65-3,90)	3,77 (2,96-4,85)	3,82 (2,81-4,04)
A’ lateral (cm/s)	3,40 (2,80-3,80)	3,95 (3,17-4,85)	4,15 (3,27-4,55)
A’ septal (cm/s)	3,25 ± 1,12	3,81 ± 1,21	3,11 ± 0,76
E’/A’	1,34 ± 0,39	0,95 ± 0,25*	1,05 ± 0,35

Dados expressos em média ± desvio-padrão ou mediana e percentis. IM-SED: infarto do miocárdio sedentário; IM-EA: infarto do miocárdio e exercício aeróbico; n: número de animais; Mitral E: velocidade de pico do influxo mitral diastólico inicial; Mitral A: velocidade de pico do influxo mitral diastólico tardio; TRIV: tempo de relaxamento isovolumétrico; E’: pico da velocidade de deslocamento diastólico inicial do anel mitral; A’: pico da velocidade de deslocamento diastólico tardio do anel mitral. ANOVA e teste de Bonferroni ou Kruskal-Wallis e Dunn;

* p<0,05 vs Sham.

### Capacidade funcional

**Figura 2:**
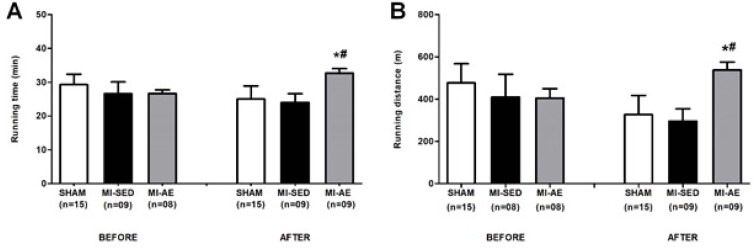
Capacidade funcional avaliada pelo teste de esforço máximo. Tempo de corrida (A) antes e depois do exercício; distância percorrida (B) antes e depois do exercício. IM-SED: infarto do miocárdio sedentário; IM-EA: infarto do miocárdio e exercício aeróbico; n: número de animais. Dados expressos em média ± desvio-padrão; ANOVA e Bonferroni; *p<0,05 vs Sham; #p<0,05 vs IM-SED.

A capacidade funcional não diferiu entre os grupos antes do exercício. Ao final do experimento, a capacidade funcional foi melhor no IM-EA do que nos demais grupos (Figura 2).

### Estudo morfométrico

**Figura 3:**
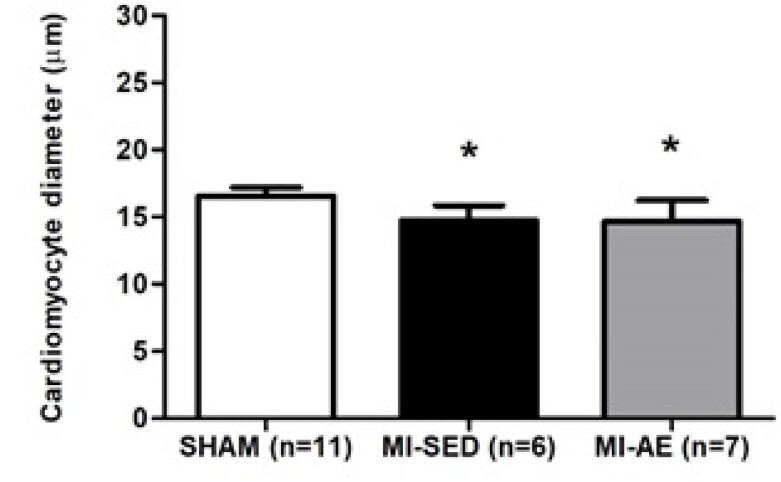
Diâmetros dos cardiomiócitos. IM-SED: infarto do miocárdio sedentário; IM-EA: infarto do miocárdio e exercício aeróbico; n: número de animais. Dados expressos em média ± desvio-padrão; ANOVA e Bonferroni; *p<0,05 vs SHAM.

O diâmetro dos cardiomiócitos foi menor nos grupos com infarto do que no Sham (Figura 3).

### Expressão gênica

A expressão gênica das subunidades NADPH oxidase NOX2, NOX4, p22^phox^ e p47^phox^ não diferiu entre os grupos (Tabela 5).

**Tabela 5:** Expressão gênica de subunidades do complexo NADPH oxidase

Gene	SHAM (n=9)	IM-SED (n=5)	IM-EA (n=5)
Nox2	1,00 ± 0,56	0,83 ± 0,34	1,07 ± 0,26
Nox4	0,99 (0,62-1,20)	1,38 (0,60-1,95)	1,36 (0,79-1,40)
p22^phox^	1,00 ± 0,35	1,12 ± 0,51	1,16 ± 0,18
p47^phox^	1,00 ± 0,56	0,83 ± 0,34	1,07 ± 0,26

Dados expressos em média ± desvio-padrão ou mediana e percentis. IM-SED: infarto do miocárdio sedentário; IM-EA: infarto do miocárdio e exercício aeróbico; n: número de animais; ANOVA e teste de Bonferroni ou Kruskal-Wallis e Dunn; p>0,05.

## Discussão

Neste estudo, avaliamos os efeitos do exercício físico aeróbico na capacidade funcional, remodelação cardíaca e expressão gênica das subunidades da NADPH oxidase em corações de ratos com pequeno IM.

Modelos experimentais de roedores com IM tem sido amplamente utilizados para investigar a fisiopatologia e o tratamento da remodelação cardíaca e insuficiência cardíaca.^26^^,^^27^ No entanto, como a anatomia da circulação coronária de um rato não é uniforme, a ligadura da artéria coronária leva a uma ampla gama de tamanhos de infarto, remodelação e disfunção do VE.^22^ Portanto, uma característica essencial dos estudos que visam estabelecer estratégias terapêuticas é avaliar animais com infarto de tamanhos comparáveis. Portanto, a avaliação ecocardiográfica do tamanho do IM e do grau de lesão cardíaca antes de iniciar estratégias terapêuticas deveria ser obrigatória.

Observamos anteriormente que o tamanho mínimo do infarto para induzir anormalidades estruturais, funcionais e clínicas era de 36%, 38% e 40% da área total do VE, respectivamente.^28^ Portanto, não esperávamos encontrar alterações cardíacas consideráveis na avaliação de ratos com IM menor de 30%. No entanto, este estudo mostrou que, ao final do período experimental, os grupos infartados apresentavam aumento do diâmetro diastólico e sistólico do VE, diâmetro do AE e massa do VE, com disfunção sistólica caracterizada por redução da fração de encurtamento endocárdico e alteração da área fracionada, bem como aumento do índice Tei. Exceto no que diz respeito à redução septal e onda E’ média, a função diastólica não diferiu entre os grupos infartados e Sham. Nossos dados, portanto, mostram que a remodelação cardíaca com dilatação das câmaras cardíacas esquerdas, e a disfunção sistólica do VE pode ser bem caracterizada em ratos com área de infarto pequena.

O fato do peso corporal não diferir entre os grupos reforça o leve grau de lesão miocárdica. A caquexia cardíaca é caracterizada por uma redução significativa no peso corporal,^29^^,^^30^ e pode ser encontrada em ratos que apresentam grandes áreas de infarto.^22^

Neste estudo, foi utilizado um protocolo de exercício aeróbico de intensidade moderada adaptado de estudos publicados anteriormente.^17^ A velocidade máxima de corrida foi estabelecida para cada rato de acordo com sua capacidade funcional, avaliada por teste de esforço máximo em esteira no início e no meio do protocolo.^15^ Ao final do experimento, notamos que o exercício era seguro e o grupo IM-EA atingiu um tempo de esteira e distância percorrida maiores do que os grupos IM-SED e Sham. Há muito tempo se sabe que o exercício aeróbico melhora a capacidade funcional na insuficiência cardíaca tanto animal quanto humana.^31^ Os resultados do grupo Sham também destacaram uma capacidade funcional reduzida causada pelo estilo de vida sedentário.

Apesar de melhorar o desempenho funcional, os efeitos do exercício aeróbico na remodelação cardíaca não foram substanciais em ratos com IM pequeno. Como um achado comum em ratos IM é a diminuição na espessura relativa da parede do VE,^22^ podemos concluir que o exercício foi útil na preservação da geometria do VE, pois a relação entre a espessura da parede posterior diastólica e o diâmetro diastólico do VE foi reduzida no IM-SED e preservada no IM-EA.

Entre as várias alterações induzidas pelo IM, o aumento do estresse oxidativo tem papel importante na progressão da remodelação cardíaca.^5^ Neste estudo, a expressão gênica das subunidades do complexo NADPH oxidase NOX2, NOX4, p22^phox^ e p47^phox^ não diferiu entre os grupos, o que sugere que essa importante fonte de geração de espécies reativas de oxigênio^4^ não esteve envolvida na remodelação cardíaca observada em ratos com infarto pequeno. Foi observado aumento da expressão gênica de NOX2 e NOX4 em roedores com IM de tamanhos grandes.^32^ Uma limitação deste estudo é que avaliamos o complexo NADPH oxidase por meio da análise da expressão gênica de suas subunidades. Portanto, estudos adicionais são necessários para avaliar a atividade do complexo NADPH oxidase.

Uma vez que a transição da disfunção VE compensada para a insuficiência cardíaca é encontrada principalmente em corações com infarto transmural grande,^22^ a maioria dos autores avaliou os efeitos do exercício em corações com grandes infartos,^8^^,^^10^^,^^33^^,^^34^ e a maioria desses estudos mostrou efeitos favoráveis do exercício aeróbico na remodelação cardíaca induzida pelo IM.^8^^,^^10^^,^^33^ Apenas alguns pesquisadores analisaram os efeitos do exercício no coração de ratos com IM pequeno.^35^^,^^36^ Ao iniciarem o exercício dentro de quatro semanas após a indução do IM, esses autores observaram efeitos benéficos do exercício físico.^35^^,^^36^ Neste estudo, mostramos pela primeira vez que o exercício aeróbico tardio, iniciado três meses após o IM, quando a remodelação cardíaca está estável, atenua as alterações da geometria cardíaca em ratos com pequeno infarto. Nosso estudo, portanto, reforça o conceito de benefício potencial da reabilitação cardíaca após síndromes coronarianas agudas, independentemente do grau de lesão cardíaca.^37^

## Conclusão

Em conclusão, o IM pequeno altera as estruturas cardíacas e a função sistólica do VE. Exercício físico aeróbico tardio melhora a capacidade funcional e atenua a alteração da geometria do VE. A expressão gênica das subunidades da NADPH oxidase não está envolvida na remodelação cardíaca e não é modulada pelo exercício aeróbico em ratos com infarto do miocárdio pequeno.
