# Supplementary material for: Efeitos do Exercício Aeróbico Tardio na Remodelação Cardíaca de Ratos com Infarto do Miocárdio Pequeno
Source: Arq Bras Cardiol. 2021 Apr 8;116(4):784–92. [Article in Portuguese] doi: 10.36660/abc.20190813 (PMC8121407; doi:10.36660/abc.20190813)
Supplement: Translation

# Translation: Effects of Late Aerobic Exercise on Cardiac Remodeling of Rats with Small-Sized Myocardial Infarction

**Authors:** Lidiane M. Souza, Marina P. Okoshi, Mariana J. Gomes, Mariana Gatto, Eder A. Rodrigues, Thierres H. D. Pontes, Felipe C. Damatto, Leiliane R. S. Oliveira, Patrícia Aparecida Borim, Aline R. R. Lima, Leonardo A. M. Zornoff, Katashi Okoshi, Luana U. Pagan

**Affiliations:** 1 BotucatuSP Brazil Faculdade de Medicina de Botucatu, Universidade Estadual Paulista “Júlio de Mesquita Filho” (UNESP), Botucatu, SP – Brazil.

**Keywords:** Exercise, Physical Exercise, Ventricular Dysfunction, Myocardial Infarction, Rats, Ventricular Remodeling, Echocardiography/methods, NADPH Oxidase

## Abstract

**Background:**

Physical exercise has been considered an important non-pharmacological therapy for the prevention and treatment of cardiovascular diseases. However, its effects on minor cardiac remodeling are not clear.

**Objective:**

To evaluate the influence of aerobic exercise on the functional capacity, cardiac structure, left ventricular (LV) function, and gene expression of NADPH oxidase subunits in rats with small-sized myocardial infarction (MI).

**Methods:**

Three months after MI induction, Wistar rats were divided into three groups: Sham; sedentary MI (MI-SED); and aerobic exercised MI (MI-AE). The rats exercised on a treadmill three times a week for 12 weeks. An echocardiogram was performed before and after training. The infarction size was evaluated by histology, and gene expression was assessed by RT-PCR. The significance level for statistical analysis was set at 5%.

**Results:**

Rats with MI lower than 30% of the LV total area were included in the study. Functional capacity was higher in MI-AE than in Sham and MI-SED rats. The infarction size did not differ between groups. Infarcted rats had increased LV diastolic and systolic diameter, left atrial diameter, and LV mass, with systolic dysfunction. Relative wall thickness was lower in MI-SED than in the MI-AE and Sham groups. Gene expression of the NADPH oxidase subunits NOX2, NOX4, p22^phox^, and p47^phox^ did not differ between groups.

**Conclusion:**

Small-sized MI changes cardiac structure and LV systolic function. Late aerobic exercise is able to improve functional capacity and cardiac remodeling by preserving the left ventricular geometry. NADPH oxidase subunits gene expression is not involved in cardiac remodeling or modulated by aerobic exercise in rats with small-sized MI.

## Introduction

Cardiovascular diseases are a leading cause of death worldwide; in this class of diseases, myocardial infarction (MI) is the main cause of morbidity and mortality.^1^

Acute MI leads to cardiac remodeling, which is defined as abnormalities in genome expression resulting in molecular, cellular and interstitial changes that manifest clinically as alterations in heart size, shape and function.^2^ Oxidative stress, characterized by an imbalance between reactive oxygen species production and antioxidant systems, is often observed during cardiac remodeling.^3^ The nicotinamide adenine dinucleotide phosphate (NADPH) oxidase complex, an important source of cellular reactive oxygen species production,^4^ is usually increased after MI.^5^

In recent decades, physical exercise has emerged as an important non-pharmacological therapy for preventing and treating several cardiovascular diseases.^6^ Aerobic exercise has been the focus of many studies for attenuating MI-induced cardiac remodeling and improving functional capacity and quality of life.^7^^–^^10^

Animal MI models are widely used for studying the pathophysiology and treatment of cardiac remodeling. Most studies evaluating the effects of exercise on post-MI cardiac changes have used rodents with large infarcted areas, usually more than 30% of the total left ventricle (LV) area.^8^^,^^11^^–^^14^ However, it is not clear yet whether aerobic exercise is useful to attenuate cardiac changes following smaller-size LV infarction. In this study we aimed to evaluate the influence of aerobic physical exercise on functional capacity, cardiac structures, LV function, and NADPH oxidase subunit gene expression in rodents with small-sized MI.

## Materials and methods

### Experimental animals

Male Wistar rats weighing 200-250 g were purchased from the Central Animal House, Botucatu Medical School, UNESP. All animals were kept in a temperature-controlled room at 24 ± 2º C and put on a 12-hour light/dark cycle in collective cages (three per cage). Food and water were supplied *ad libitum*.

All experiments and procedures were approved by the Animal Experimentation Ethics Committee of the Botucatu Medical School, UNESP, SP, Brazil, which follows the guidelines established by the Guide for the Care and Use of Laboratory Animals published by the U.S. National Institutes of Health, and Brazilian College for Animal Experimentation (protocol number 1237/2017).

MI was induced by ligating the left anterior descending coronary artery according to a previously described method.^3^^,^^14^ Briefly, 60 rats were anesthetized with ketamine (60 mg/kg) and xylazine hydrochloride (1 mg/kg) and subjected to left lateral thoracotomy. After exteriorization of the heart, the left atrium was retracted to facilitate ligation of the coronary artery with a 5-0 monofilament nylon suture between the pulmonary outflow tract and the left atrium. The heart was then placed back in the thorax, the lungs inflated with positive pressure, and the thoracotomy was closed. Fifteen sham-operated animals were used as controls.

Three months later, the rats that survived were subjected to transthoracic echocardiogram and exercise testing and then assigned to three groups: Sham (n=15); sedentary MI (MI-SED, n=22) and aerobic exercised MI (MI-AE, n=21) for three months. Seventeen infarcted rats (28%) died during surgery or in the post-operative period. Initial echocardiogram results were used to assure that sedentary and exercise MI groups had the same degree of cardiac injury. At the end of the experimental period, the animals were again subjected to echocardiogram and exercise testing, and euthanized the next day. Previous studies have shown that the inclusion of 10 to 15 animals per group is sufficient to show differences in cardiac remodeling when comparing infarcted and Sham rats.^3^^,^^14^

### Exercise testing

Functional capacity was evaluated before, 45 days after initiating exercise, and at the end of the experiment. Rats underwent 5 min/day an adaption to test environment for one week before evaluation. Each animal was tested individually. The test consisted of an initial 5-minute warm-up at 5 m/min on a treadmill. The rats were then subjected to exercise at 8 m/min followed by increments of 3 m/min every 3 minutes until exhaustion. Exhaustion was determined when the animal refused to run even after electric stimulation or was unable to coordinate steps.^15^^,^^16^ The maximum running speed was recorded and total distance was calculated. Exercise test results from 45-day training were used to adjust exercise intensity.

### Exercise training protocol

Exercise was performed on a treadmill three days/week for three months. There was an adaptation period, with a gradual increase in speed and exercise duration. Speed from the 1^st^ to the 5^th^ week was 5, 7.5, 10, 12 and 15 m/min. Exercise duration from the 1^st^ to the 5^th^ week was 10, 15, 25, 30 and 40 minutes. From the 6^th^ week on, each session consisted of 40 minutes of running at 60% of maximum velocity reached in the treadmill exercise test. The protocol was adapted from Moreira et al.^17^ After 45 days of aerobic exercise training, animals had their running performance reevaluated as to adjust exercise intensity.

### Echocardiography

Cardiac structures and LV function were evaluated by transthoracic echocardiogram and tissue Doppler imaging using a commercially available echocardiograph (General Electric Medical Systems, Vivid S6 model, Tirat Carmel, Israel) equipped with a 5–11.5 MHz multifrequency transducer, as previously described.^18^^–^^20^ The animals were anesthetized with ketamine (50 mg/kg) and xylazine hydrochloride (1 mg/kg *i.p.*), and placed in left lateral decubitus. All cardiac structures were manually measured by the same observer (KO). Results were the mean of at least five cardiac cycles on M-mode tracings. The following structural variables were measured: left atrium diameter (LA), LV diastolic and systolic diameters (LVDD and LVSD, respectively), LV diastolic posterior wall thickness (DPWT) and aortic diameter (AO). Left ventricular mass (LVM) was calculated using the formula [(LVDD + DPWT + DSWT)^3^− LVDD^3^] × 1.04. LV relative wall thickness (RWT) was calculated with the formula 2 × DPWT/LVDD. Systolic function was assessed by the following parameters: endocardial fractional shortening (EFS), posterior wall shortening velocity (PWSV), fractional area change (FAC), myocardial performance index (Tei index), and systolic velocity of the mitral annulus (S’ wave) obtained by tissue Doppler imaging. The diastolic function was analyzed by early and late diastolic mitral inflow velocities (E and A waves), E/A ratio, isovolumetric relaxation time (IVRT), early diastolic (E’) and late diastolic (A’) velocity of the mitral annulus (arithmetic average travel speeds of the lateral and septal walls), and E/E’ ratio.

### Collection of tissues for analysis

One day after final echocardiogram, the animals were weighed, anesthetized with intraperitoneal sodium thiopental (180 mg/kg) and euthanized. Their hearts were removed by thoracotomy. The lung, atria and ventricles were dissected and weighed. Fragments of LV were frozen in liquid nitrogen and stored at −80 °C for posterior analysis.

### Morphologic study

LV samples were fixed in a 10% buffered formalin solution for 24 hours, then washed in water and transferred to a solution with ethanol, according to a previously described method.^21^

To calculate infarction size, the LV was cut at a distance of 5 to 6 mm from the apex.^22^ Heart slices were stained with picrosirius red (PSR) and examined under a compound microscope (Leica DM LS; Nussloch, Germany) coupled to a computerized imaging analysis system (Media Cybernetics, Silver Spring, Maryland, USA).^23^ The infarction size was calculated by dividing the sum of endocardial and epicardial infarcted ventricular lengths by the sum of the total (infarcted and viable myocardium) endocardial and epicardial ventricular circumferences.^14^ Values were expressed as percentage of the total LV area. Only rats with small-sized MI (<30% of total LV area) at histological evaluation were included in the study.

Cardiomyocyte diameters were assessed in LV transverse sections stained with hematoxylin-eosin. The smallest diameter of at least 50 cardiac fibers with the nucleus clearly identified was measured.^24^

### Gene expression of NADPH oxidase subunits

Gene expression of NADPH oxidase subunits NOX2, NOX4, p22^phox^, and p47^phox^ and reference genes was analyzed by Real-Time Quantitative Reverse Transcription-Polymerase Chain Reaction (RT-PCR), as previously described.^25^ Total RNA was extracted from LV samples with TRIzol Reagent (Invitrogen Life Technologies, Carlsbad, CA, USA) and treated with DNase I (Invitrogen Life Technologies). One microgram of RNA was reverse-transcribed using a High-Capacity cDNA Reverse Transcription kit, according to standard methods (Applied Biosystems, Foster City, CA, USA). Aliquots of cDNA were then submitted to real-time PCR using a customized assay containing sense and antisense primers and Taqman (Applied Biosystems, Foster City, CA, USA) probes specific to each gene: NOX2 (Rn00576710 m1), NOX4 (Rn00585380 m1), p22^phox^ (Rn00577357 m1), and p47^phox^ (Rn00586945 m1). Amplification and analysis were performed using the Step One Plus™ Real-Time PCR System (Applied Biosystems, Foster City, CA, USA). Expression data were normalized to reference gene expressions: cyclophilin (Rn00690933 m1) and GAPDH (Rn01775763 g1). Reactions were performed in triplicate and expression levels calculated based on the CT comparative method (2^−ΔΔCT^).

### Statistical analyzes

Data normality was evaluated by the Shapiro-Wilk test. Comparisons between groups were performed by one-way analysis of variance (ANOVA), followed by the Bonferroni test for parametric variables, which are expressed as mean ± standard deviation. Non-parametric variables were compared using the Kruskal-Wallis test followed by Dunn's test, being expressed as median and percentiles. Infarction size was compared by the unpaired *Student t* test. Statistical analyzes were performed on the SigmaStat 12.0 software. The significance level was set at 5%.

## Results

### Experimental groups and anatomical parameters

At the beginning of the exercise protocol, the Sham group had 15 animals, MI-SED had 22, and MI-AE had 21. After histologic analysis, the rats with infarction size ≥ 30% of total LV area (9 in MI-SED and 9 in MI-AE group) were excluded from the study. Only one rat from MI-SED died during the exercise protocol. Anatomical parameters are shown in Table 1. Final body weight did not differ between groups. Atria and right ventricle (RV) weights were higher in MI-AE than in Sham group. No differences between MI-AE and MI-SED groups were found.

**Table 1:** Anatomical data

	SHAM (n=15)	MI-SED (n=12)	MI-AE (n=12)
BW (g)	536 ± 29.7	537 ± 66.8	529 ± 44.7
LV (g)	0.90 (0.87-0.97)	0.99 (0.93-1.03)	0.99 (0.90-1.11)
LV/BW (g/kg)	1.73 ± 0.10	1.90 ± 0.19	1.88 ± 0.23
RV (g)	0.23 ± 0.03	0.26 ± 0.04	0.29 ± 0.05*
RV/BW (g/kg)	0.43 ± 0.05	0.48 ± 0.07	0.54 ± 0.08*
Atrial weight (g)	0.10 (0.08-0.11)	0.13 (0.10-0.13)	0.13 (0.11-0.14)*
Atrial/BW (g/kg)	0.19 (0.15-0.22)	0.22 (0.19-0.24)	0.27 (0.22-0.28)*
Lung/BW (g/kg)	3.60 (3.19-3.70)	3.43 (3.09-3.72)	3.66 (3.58-4.13)

Data are expressed as mean ± standard deviation or median and percentiles. MI-SED: sedentary myocardial infarction; MI-AE: aerobic exercise myocardial infarction; n: number of animals; BW: body weight; LV: left ventricle weight; RV: right ventricle weight. ANOVA and Bonferroni or Kruskal-Wallis and Dunn's test;

* p<0.05 vs. Sham.

Infarction size, assessed by LV histological analysis, did not differ between infarcted groups (MI-SED 18.7 ± 6.41; MI-AE 23.6 ± 6.14% of total LV area; p>0.05; Figure 1).

**Figure 1:** Representative histological photos of picrosirius red-stained portions of left ventricles from the groups SHAM, sedentary myocardial infarction (MI-SED), and aerobic exercise myocardial infarction (MI-AE).

### Echocardiographic evaluation

Before exercise, there were no differences in echocardiographic parameters between MI-AE and MI-SED groups (data not shown). Final echocardiographic structural data are listed in Table 2. Both infarcted groups had higher LV systolic and diastolic diameters, left atrial diameter, and LV mass compared to the Sham group. LV diastolic posterior wall thickness was higher in MI-AE than in Sham, and relative wall thickness was lower in MI-SED than in MI-AE and Sham groups. LV systolic function is shown in Table 3. Infarcted groups had lower fractional area change and endocardial fractional shortening, as well as higher Tei index when compared to Sham. LV diastolic function is presented in Table 4. E’ (average and septal) wave was lower in both infarcted groups as related to the Sham group. MI-AE had lower E/A ratio than Sham. E’/A’ ratio was lower in MI-SED than in Sham. No differences were observed between exercised and sedentary infarcted groups.

**Table 2:** Echocardiographic structural data

	SHAM (n=15)	MI-SED (n=10)	MI-AE (n=12)
HR (bpm)	267 ± 32.9	278 ± 19.7	290 ± 28.7
LVDD (mm)	8.19 ± 0.44	9.99 ± 0.81*	9.93 ± 0.98*
LVSD (mm)	4.13 (3.96-4.30)	7.16 (6.60-8.21)*	7.25 (6.73-8.16)*
DPWT (mm)	1.42 (1.40-1.45)	1.53 (1.45-1.61)	1.67 (1.58-1.85)*
AO (mm)	4.20 ± 0.15	4.12 ± 0.22	4.13 ± 0.25
LA (mm)	5.68 ± 0.42	6.71 ± 0.75*	6.97 ± 1.07*
LA/AO	1.37 (1.30-1.42)	1.64 (1.47-1.79)*	1.66 (1.47-1.82)*
LVDD/BW (mm/kg)	15.2 (14.8-16.3)	17.9 (16.9-20.3)*	18.5 (17.8-20.1)*
LA/BW (mm/kg)	10.7 ± 0.95	12.4 ± 1.42*	13.5 ± 2.46*
LVM (g)	0.84 (0.76-0.91)	1.29 (1.17-1.43)*	1.27 (1.22-1.63)*
LVMI (g/kg)	1.57 (1.46-1.70)	2.32 (2.12-2.63)*	2.44 (2.31-3.08)*
RWT	0.35 ± 0.02	0.31 ± 0.02*	0.35 ± 0.04#
% area MI	No infarction	26.23 ± 5.77	27.62 ± 7.67

Data are expressed as mean ± standard deviation or median and percentiles. MI-SED: sedentary myocardial infarction; MI-AE: aerobic exercise myocardial infarction; n: number of animals; HR: heart rate; LVDD and LVSD: left ventricular diastolic and systolic diameters respectively; DPWT: left ventricular diastolic posterior wall thickness; AO: aorta diameter; LA: left atrial diameter; BW: body weight; LVM: left ventricular mass; LVMI: left ventricular mass index; RWT: relative wall thickness. % area MI: percentage of myocardial infarcted area. ANOVA and Bonferroni or Kruskal-Wallis and Dunn's test;

* p<0.05 vs Sham;

# p<0.05 vs MI-SED.

**Table 3:** Echocardiographic parameters of left ventricular systolic function

	SHAM (n=15)	MI-SED (n=10)	MI-AE (n=12)
EFS (%)	49.7 ± 3.40	27.0 ± 5.23*	26.6 ± 7.91*
PWSV (mm/s)	42.1 ± 5.66	35.9 ± 5.37	38.7 ± 9.28
FAC (%)	67.3 ± 5.07	41.1 ± 9.95*	37.6 ± 10.5*
Tei index	0.46 ± 0.06	0.58 ± 0.12*	0.58 ± 0.15*
S’ average (cm/s)	3.55 ± 0.40	3.15 ± 0.34	3.20 ± 0.47

Data are expressed as mean ± standard deviation. MI-SED: sedentary myocardial infarction; MI-AE: aerobic exercise myocardial infarction; n: number of animals; EFS: endocardial fractional shortening; PWSV: posterior wall shortening velocity; Tei index: myocardial performance index; S’ average: mean maximum systolic displacement velocities for lateral and septal walls of the mitral annulus assessed by tissue Doppler imaging. ANOVA and Bonferroni;

* p<0.05 vs Sham.

**Table 4:** Echocardiographic parameters of left ventricular diastolic function

	SHAM (n=15)	MI-SED (n=10)	MI-AE (n=12)
Mitral E (cm/s)	77.0 (71.0-85.0)	72.5 (69.3-79.5)	75.5 (72.8-78.0)
Mitral A (cm/s)	49.1 ± 12.2	54.3 ± 11.9	59.9 ± 16.8
E/A	1.71 (1.42-1.79)	1.32 (1.26-1.49)	1.23 (1.07-1.35)*
IVRT (m/s)	26.5 ± 3.42	29.7 ± 5.75	28.0 ± 3.79
E’ average (cm/s)	4.20 ± 0.63	3.52 ± 0.62*	3.58 ± 0.50*
E’ lateral (cm/s)	4.16 ± 0.73	3.20 ± 0.56*	3.24 ± 0.74*
E’ septal (cm/s)	4.24 ± 0.61	3.84 ± 0.88	3.92 ± 0.79
E/E’ average	19.1 ± 2.65	21.8 ± 3.47	21.6 ± 2.35
A’ average (cm/s)	3.05 (2.65-3.90)	3.77 (2.96-4.85)	3.82 (2.81-4.04)
A’ lateral (cm/s)	3.40 (2.80-3.80)	3.95 (3.17-4.85)	4.15 (3.27-4.55)
A’ septal (cm/s)	3.25 ± 1.12	3.81 ± 1.21	3.11 ± 0.76
E’/A’	1.34 ± 0.39	0.95 ± 0.25*	1.05 ± 0.35

Data are expressed as mean ± standard deviation or median and percentiles. MI-SED: sedentary myocardial infarction; MI-AE: aerobic exercise myocardial infarction; n: number of animals; Mitral E: peak velocity of early-diastolic mitral inflow; Mitral A: peak velocity of late-diastolic mitral inflow; IVRT: isovolumetric relaxation time; E’: peak initial diastolic displacement velocity of the mitral annulus; A’: peak late diastolic displacement velocity of the mitral annulus. ANOVA and Bonferroni or Kruskal-Wallis and Dunn's test;

* p<0.05 vs Sham.

### Functional capacity

Functional capacity did not differ between groups before exercise. At the end of the experiment, functional capacity was better in MI-AE than in the other groups (Figure 2).

**Figure 2:** Functional capacity evaluated by maximal exercise test. Running time (A) before and after exercise; running distance (B) before and after exercise. MI-SED: sedentary myocardial infarction; MI-AE: aerobic exercise myocardial infarction; n: number of animals. Data are expressed as mean ± standard deviation; ANOVA and Bonferroni; *p<0.05 vs SHAM; #p<0.05 vs MI-SED.

### Morphometric study

Cardiomyocyte diameter was smaller in infarcted groups than in Sham (Figure 3).

**Figure 3:** Cardiomyocyte diameters. MI-SED: sedentary myocardial infarction; MI-AE: aerobic exercise myocardial infarction; n: number of animals. Data are expressed as mean ± standard deviation; ANOVA and Bonferroni; *p<0.05 vs SHAM.

### Gene expression

Gene expression of NADPH oxidase subunits NOX2, NOX4, p22^phox^, and p47^phox^ did not differ between groups (Table 5).

**Table 5:** Gene expression of NADPH oxidase complex subunits

Gene	SHAM (n=9)	MI-SED (n=5)	MI-AE (n=5)
Nox2	1.00 ± 0.56	0.83 ± 0.34	1.07 ± 0.26
Nox4	0.99 (0.62-1.20)	1.38 (0.60-1.95)	1.36 (0.79-1.40)
p22phox	1.00 ± 0.35	1.12 ± 0.51	1.16 ± 0.18
p47phox	1.00 ± 0.56	0.83 ± 0.34	1.07 ± 0.26

Data are expressed as mean ± standard deviation or median and percentiles. MI-SED: sedentary myocardial infarction; MI-AE: aerobic exercise myocardial infarction; n: number of animals; ANOVA and Bonferroni or Kruskal-Wallis and Dunn's test; p>0.05.

## Discussion

In this study, we evaluated the effects of aerobic physical exercise on functional capacity, cardiac remodeling and gene expression of NADPH oxidase subunits in small-sized MI rat hearts.

The rodent experimental MI model has been widely used to investigate the pathophysiology and treatment of cardiac remodeling and heart failure.^26^^,^^27^ However, as a rat's coronary circulation anatomy is not uniform, coronary artery ligation leads to a wide range of infarct sizes, cardiac remodeling, and LV dysfunction.^22^ Therefore, an essential feature of studies aimed to establish therapeutic strategies is to evaluate animals with comparable infarction sizes. Thus, echocardiographic assessment of MI size and cardiac injury degree before initiating therapeutic strategies should be mandatory.

We have previously observed that the minimum infarct size for inducing structural, functional, and clinical abnormalities was 36%, 38%, and 40% of the total LV area, respectively.^28^ We therefore did not expect to find considerable cardiac changes by evaluating rats with MI sizes below 30%. However, this study showed that, at the end of the experimental period, infarcted groups presented increased LV diastolic and systolic diameter, left atrial diameter, and LV mass, with systolic dysfunction characterized by reduced endocardial fractional shortening and fractional area change, as well as increased Tei index. Except for reduced septal and average E’ wave, the diastolic function did not differ between infarcted and Sham groups. Our data therefore showed that cardiac remodeling with left cardiac chambers dilation and LV systolic dysfunction can be well characterized in rats with small-sized infarction area.

The fact that body weight did not differ between groups reinforces the slight degree of myocardial injury. Cardiac cachexia is characterized by a significant reduction in body weight,^29^^,^^30^ and can be found in post-infarction rats with large infarction areas.^22^

In this study, we used a moderate intensity aerobic exercise protocol adapted from previously published studies.^17^ Maximum running velocity was established for each rat according to its functional capacity, evaluated by maximum effort test performed on a treadmill at the beginning and middle of the exercise protocol.^15^ At the end of the experiment, we noted that exercise was safe and the MI-AE group attained a higher treadmill time and distance run than MI-SED and Sham groups. Aerobic exercise has long been shown to improve functional capacity in both animal and human heart failure.^31^ The Sham rat results also underlined a reduced functional capacity caused by sedentary lifestyle.

Despite improving functional performance, the effects of aerobic exercise on cardiac remodeling were not substantial in small-sized MI rats. As a common finding in MI rats is a decrease in LV relative wall thickness,^22^ we may conclude that exercise was helpful in preserving LV geometry, as the relationship between LV diastolic posterior wall thickness and LV diastolic diameter was reduced in MI-SED and preserved in the MI-AE group.

Among various MI-induced alterations, increased oxidative stress plays an important role in cardiac remodeling progression.^5^ In this study, gene expression of NADPH oxidase complex subunits NOX2, NOX4, p22^phox^, and p47^phox^ did not differ between groups, which suggests that this important source of reactive oxygen species generation^4^ was not involved in the cardiac remodeling observed in rats with small-sized infarction. Increased gene expression of NOX2 and NOX4 has been observed in large-sizes MI rodents.^32^ One limitation of this study is that we have evaluated NADPH oxidase complex by analyzing the gene expression of its subunits. Therefore, additional studies are needed to assess the activity of the NADPH oxidase complex.

Since transition from compensated LV dysfunction to heart failure is mainly found in hearts with large transmural infarction,^22^ most authors have evaluated the effects of exercise on hearts with large infarct sizes^8^^,^^10^^,^^33^^,^^34^ and most studies have shown favorable effects of aerobic exercise on MI-induced cardiac remodeling.^8^^,^^10^^,^^33^ Only a few researchers have analyzed the cardiac effects of exercise in rats with small-sized MI.^35^^,^^36^ By initiating exercise within four weeks post MI induction, these authors have observed beneficial cardiac effects of physical exercise.^35^^,^^36^ In this study we showed for the first time that late aerobic exercise, initiated three months after MI, when cardiac remodeling is stable, attenuates cardiac geometry changes in rats with small-sized infarction. Our study therefore reinforces the concept of potential benefit from cardiac rehabilitation after acute coronary syndromes, regardless of cardiac injury degree.^37^

## Conclusion

In conclusion, small-sized MI changes cardiac structures and the left ventricular systolic function. Late aerobic physical exercise improves functional capacity and attenuates left ventricular geometry change. NADPH oxidase subunits gene expression is not involved in cardiac remodeling nor is modulated by aerobic exercise in rats with small-sized MI.
